# Metabonomics Analysis of Plasma Reveals the Lactate to Cholesterol Ratio as an Independent Prognostic Factor of Short-Term Mortality in Acute Heart Failure

**DOI:** 10.1371/journal.pone.0060737

**Published:** 2013-04-03

**Authors:** Franck Desmoulin, Michel Galinier, Charlotte Trouillet, Matthieu Berry, Clément Delmas, Annie Turkieh, Pierre Massabuau, Heinrich Taegtmeyer, Fatima Smih, Philippe Rouet

**Affiliations:** 1 INSERM I2MC, UMR 1048, Université UPS, Equipe «Obésité et insuffisance cardiaque: approches moléculaires et cliniques », Toulouse, France; 2 CHU de Rangueil, Service de Cardiologie A, Toulouse, France; 3 Division of Cardiology, Department of Internal Medicine, The University of Texas Medical School at Houston, Houston, Texas, United States of America; Virginia Commonwealth University, United States Of America

## Abstract

**Objective:**

Mortality in heart failure (AHF) remains high, especially during the first days of hospitalization. New prognostic biomarkers may help to optimize treatment. The aim of the study was to determine metabolites that have a high prognostic value.

**Methods:**

We conducted a prospective study on a training cohort of AHF patients (n = 126) admitted in the cardiac intensive care unit and assessed survival at 30 days. Venous plasmas collected at admission were used for *^1^H NMR* **–** *based* metabonomics analysis. Differences between plasma metabolite profiles allow determination of discriminating metabolites. A cohort of AHF patients was subsequently constituted (n = 74) to validate the findings.

**Results:**

Lactate and cholesterol were the major discriminating metabolites predicting 30-day mortality. Mortality was increased in patients with high lactate and low total cholesterol concentrations at admission. Accuracies of lactate, cholesterol concentration and lactate to cholesterol (Lact/Chol) ratio to predict 30-day mortality were evaluated using ROC analysis. The Lact/Chol ratio provided the best accuracy with an AUC of 0.82 (P < 0.0001). The acute physiology and chronic health evaluation (APACHE) II scoring system provided an AUC of 0.76 for predicting 30-day mortality. APACHE II score, Cardiogenic shock (CS) state and Lact/Chol ratio ≥ 0.4 (cutoff value with 82% sensitivity and 64% specificity) were significant independent predictors of 30-day mortality with hazard ratios (HR) of 1.11, 4.77 and 3.59, respectively. In CS patients, the HR of 30-day mortality risk for plasma Lact/Chol ratio ≥ 0.4 was 3.26 compared to a Lact/Chol ratio of < 0.4 (P  =  0.018). The predictive power of the Lact/Chol ratio for 30-day mortality outcome was confirmed with the independent validation cohort.

**Conclusion:**

This study identifies the plasma Lact/Chol ratio as a useful objective and simple parameter to evaluate short term prognostic and could be integrated into quantitative guidance for decision making in heart failure care.

## Introduction

Acute heart failure (AHF) is the most frequent cause of hospital admission among patients over 65 years [1] and a common presentation of patients admitted to an intensive care unit. Despite advances in treatment, morbidity and mortality of AHF remain high [2] as it is the case with the rate of rehospitalizations [3]. A survey on the quality of care among patients with heart failure in Europe has shown 13.5% mortality between admission and 12 weeks follow-up [4]. There is a need for a simple test to identify patients with the higher mortality risk in order to optimize medical care.

Evaluation of heart failure patients includes a focused history, physical examination, an electrocardiogram and an echocardiogram. Altogether, these complementary approaches are aimed at better management strategies. Measurements of the biomarker brain natriuretic peptides (BNPs) are the most commonly used HF biomarkers associated with altered hemodynamics [5]. BNPs are of both diagnostic and prognostic importance, and BNP levels at admission are significantly higher in patients who suffered a cardiac death within 3 months of hospital discharge [6]. However, blood BNPs level monitoring has limitations. Several reports have underlined their high variability despite some improvement gained with the quantification of amino-terminal pro-BNP [6], [7]. Recently, positive serum cardiac troponin I was associated with high in-hospital mortality [8], reflecting heart muscle damage. However, the quest for prognostic biomarkers is an ongoing challenge because so far no blood molecule has been identified as a marker with a significantly high level of specificity and sensibility.

For the last decade the “omics” technology has gained in popularity in screens of gene expression, proteins and metabolites [9], [10], [11], [12], [13]. The level of severity of heart disease affects the blood metabonome [14], [15]. We hypothesized that the plasma metabolome may be a predictor of short-term mortality. Thus, we conducted a study of the association of short-term outcomes with altered plasma metabolites levels on admission in hospitalized patients with acute heart failure. A ^1^H NMR-based metabonomic approach has been chosen mainly because of its capability to detect hydrophilic and lipophilic metabolites without hyphenated separation techniques. Metabolic profiling was performed at hospital admittance and led us to identify metabolites associated with the 30-day mortality outcome of patients admitted for AHF. Moreover, we sought to evaluate the 30-day predictive power of these metabolites.

## Patients and Methods

### Ethics statement

This research protocol was registered in a clinical database (ClinicalTrials.gov NCT01024049) and conform to the ethical guidelines of the 1975 Declaration of Helsinki. The protocol was approved by the institution’s human research (COSSEC) and regional ethics committee (Comité de Protection des Personnes (CPP) # DC 2008-452). Written informed consent was obtained from all participants and/or their legally authorized representatives.

### Study design

**Figure 1:**
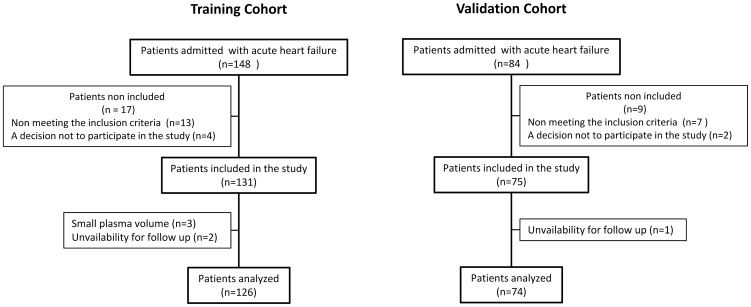
Flow-chart of the study.

We enrolled 148 plus 84 consecutive patients admitted to the cardiac intensive care unit (ICU) at Rangueil Hospital, Toulouse, France. This investigation included two independent patient groups, a training cohort and a validation cohort, which were evaluated separately. The training cohort was prospectively enrolled between July, 2008 and April, 2009, and consisted of 148 patients who were admitted with AHF. Inclusion criteria were the diagnosis of AHF based on symptoms, physical findings and echocardiography. Exclusion criteria were stroke within the last 6 months, acute liver failure within the last 6 months or known chronic hepatic failure (alanine aminotransferase or aspartate amino transférase > 5 times the upper regular limits), history for alcohol abuse or drug addiction, cancer or a diagnosis of cancer within 5 years, history of recent or current drug or alcohol abuse, participation in any clinical trial within 30 days prior to admission, hematological pathology (myelodegenerative syndrome, severe anemia (Hb < 8 g / 100 ml) or severe neutropenia (neutrophile count < 1000 cells /µl), thrombocypenia (platelet count < 7500 plts/µl). Seventeen patients met the exclusion criteria; four who refused to participate, three with history of alcohol abuse, two with stroke within last 6 months, two with acute liver failure within 6 months or known chronic liver failure, two with cancer or a diagnosis of cancer within 5 years. Baseline demographics data, admission vital signs, clinical history, assessment of cardiovascular risk factors and admission medication were recorded in a dedicated database. Biochemical data including BNP levels were obtained upon ICU admission. A chest x-ray, and electrocardiogram obtained within 6 hours of the admission. Venous blood samples were obtained during the morning within 24 hours following admission. Three patients were not analyzed because of insufficient blood volume withdrawn. A validation cohort was subsequently enrolled between May, 2010 and September, 2010 and consisted of 84 patients who were admitted with AHF. Two patients refused to participate and nine patients met the exclusion criteria. The primary endpoint was mortality at 30 days. Survival status of outpatients was obtained by telephone contact with the primary physicians or with patients directly. Two patients of the training cohort and one patient of the validation cohort were unavailable for follow up. The final study populations constituted 126 patients for the training cohort and 74 patients for the validation cohort (Figure 1). APACHE II score [16] was retrospectively evaluated with medical records of patients included to the training cohort.

### Metabonomic analysis

Venous blood samples dedicated to ^1^H-NMR-based metabonomic study were collected in Becton Dickinson Vacutainer CPT tubes with sodium heparin. Plasma was dispatched into 3×1 ml vials and stored at −80°C. One of these plasma aliquots was used for diluted plasma ^1^H NMR analysis (250 µl was diluted into 755 µl final mixture of 500 µl 0,9% saline in D_2_O and 5 µl 100 mM sodium 3-(trimethylsilyl) propionate-2,2,3,3-d4 (TSP)) immediately before the NMR spectra acquisition. The second plasma aliquot was submitted to an extraction process to isolate the hydrophilic and lipophilic plasma metabolites into two separated fractions that were analyzed serially on the day after. Simultaneous extraction of lipophilic and polar metabolites was performed with ice-cold methanol, chloroform and water (2∶2∶1.3, v/v/v) [17]. The aqueous fraction of the extract was reconstituted in 600 µl of D_2_O phosphate buffered solution with 10 µl of a 10 mM 3-(trimethylsilyl)-1-propanesulfonate sodium salt (TMPS) before NMR analysis. The organic fraction of the extract was reconstituted in 1 ml CDCl_3_ with 10 µl TCB (100 mM) and maintained under nitrogen atmosphere at −80°C until NMR analysis.

^1^H NMR spectra were recorded at 300 K on a Bruker Avance DRX 600 spectrometer operating at 600.13 MHz and equipped with a 5 mm triple axis inverse (TXI) gradient cryoprobe. Four plasma spectra were acquired for each patient: 1) spectrum of diluted plasma sample acquired with presaturation of the water signal and using one-pulse sequence, namely the Zg-spectrum; 2) spectrum of diluted plasma acquired with presaturation of the water and CPMG spin-echo sequence, namely the cpmg-spectrum; 3) spectrum of aqueous fraction of the plasma extract, namely the Aq-spectrum; 4) spectrum of the organic fraction of the plasma extract, namely the Org-spectrum. Zg-spectrum and CPMG spin-echo sequence (cpmg spectrum) with an echo loop time (2nπ) of 320 ms. A total of 64 transients were sampled with a spectral width of 12 ppm, 32 K data point on time domain (2.3 s acquisition time) and 2.5 s additional relaxation delay. Spectra of aqueous and organic fractions were serially acquired using an automatic sample changer (B-ACS 60). Spectra of aqueous fractions (Aq. spectrum) were obtained with similar parameters to the one-pulse spectrum of the diluted plasma whereas spectra of the organic fraction (Org. spectrum) were acquired with an additional delay of 4 s and without solvent suppression. ^1^H NMR spectra were processed using the TOPSPIN (version 2.1, Bruker BioSpin SA, France) and AMIX (Bruker Analytik, Rheinstetten, Germany) software packages. Typical processing parameters were 65 K zero-filling and an exponential apodizing function (0.3 Hz) applied prior to Fourier transform. Phase and base-line corrections of spectra were performed by operator and referenced with AMIX software to methyl resonance of TMPS, lactate or TCB for diluted plasma, aqueous fraction and organic fraction respectively. ^1^H NMR spectra were processed using the TOPSPIN (version 2.1, Bruker BioSpin SA, France) and AMIX (Bruker Analytik, Rheinstetten, Germany) software packages.

To perform data reduction and pattern recognition, each of the four NMR spectra obtained per patient were bucketed to obtain spectral data sets using the AMIX (Bruker Analytik, Rheinstetten, Germany) software package. The generated variables were identified with the central chemical shift value of the bins as suffix and Zg, cpmg, Aq or Org as prefixes. This raw data matrix was exported into the SIMCA-P+ (version 12.0, Umetrics, Umeå, Sweden) software to be separately orthogonalized with an OSC filtering function [18] prior fusioned in a normalized matrix of 672 rows (X-block) and 126 lines. To maximize separation between the groups, partial least squares-discriminant analysis (PLS-DA) was performed by using survival or 30-day mortality as Y (Y-block). The statistical results obtained by PLS-DA methods are able to detect which variables in the X-block are relevant to determine the dependent variables (Y-block) by means of the variable Influence on projection parameter (IP) values. The IP values reflect, in fact, the importance of terms in the model both with respect to Y, i.e. its correlation with all the responses, and with respect to X.

### Automated lactate, cholesterol and BNP assays

Plasma concentrations of lactate and total cholesterol were determined from a third plasma aliquot. Lactate and total cholesterol concentration were determined by a routine laboratory method using a COBAS MIRA+ autoanalyzer according to the manufacturer's instructions (HORIBA ABX diagnostic, Montpellier, France). Cholesterol was measured by the CHOD-PAP method with kit A11A01634 and lactate was measured by the enzymatic colorimetric Trinder method with kit A11A01721 (HORIBA ABX diagnostic, Montpellier, France). Plasma BNP was immediately analyzed from EDTA-anticoagulated blood samples using the Centaur Bayer kit (Bayer HealthCare, France) and a Centaur (Siemens, France) hospital automat as recommended by the manufacturers. In the validation cohort, plasma concentrations of lactate and total cholesterol were independently determined on the day the blood was drawn at the Rangueil hospital Biochemistry department.

### Statistical Analysis

Unless otherwise specified, continuous variables are presented as means (±SD) and categorical variables as percentages. Each of the variables used to describe characteristics of the training cohort was examined for its univariate association with 30-day mortality. For categorical variables, a Pearson Chi-square test was used to determine the statistical significance of the association between the variable and 30-day mortality. For continuous variables, a Student’s *t-*test or Mann-Whitney rank sum test when normality test failed was used to determine the statistical significance of the association between the variable and 30-day mortality with a 2-tailed P value determination. (Medcalc, version 11.6.0.0, Medcalc software bva, Belgium). Variables (except those related to admission medication and early in-hospital management) that were significantly associated with 30-day mortality with significance level of *P* < 0.05 were selected for univariate and multivariate Cox regression. Continuous variables were dichotomized at the cutoff levels. The maximum value of the Youden index (sensitivity + specificity -1) on receiver operating characteristics analysis was used in determining the cutoff level. Separate Cox regression using the enter method was applied to evaluate the hazard ratio of covariates adjusted for age and sex. Multivariate Cox proportional hazards analysis was performed as stepwise regression (enter variable if *P* < 0.2, remove variable if *P* > 0.5) with variables that were associated with 30-day mortality with *P* < 0.05, Lact/Chol ratio, HDL, acute decompensation of CHF (ADCHF) and cardiogenic shock (CS). Survival curves were constructed using the Kaplan-Meier method and compared with the log-rank test. Comparison of the areas under ROC curves for APACHE II score and Lact/Chol ratio was performed with a non-parametric method [19].

Capability of the Lact/Chol ratio to predict 30-day mortality was further validated on the independent validation cohort with receiver operating characteristic, survival curves and Cox proportional hazards survival regression analysis.

## Results

### Demographic data and clinical characteristics

Demographic and clinical data are presented in Table 1. The patients were 69 ± 15 years old, (61% male) and the LVEF was ≤ 45% in 75% of patients. Patients had significant comorbid conditions, including hypertension (58%) coronary heart disease (52%) and diabetes mellitus (38%). Most patients were on diuretics (91%) and antiplatelet agents (71%). Twenty-eight patients (22%) died within 30 days following their admission. Clinical presentation for CS and admission diagnosis for acute decompensation of chronic heart failure (CHF) parameters were significantly higher in patients who died within 30 days than in those with favorable outcome, 79 vs 21% (*P* < 0.001) and 78 vs 53% (*P  = *0.031) respectively. Clinical presentation for pulmonary edema or hypertensive AHF, medication with beta-blockers and mean blood pressure parameters were significantly higher in patients with favorable outcome on day 30 following admission than in those who died within 30 days, 72 vs 7 (P < 0.001), 39 vs 10 (*P  = * 0.008) and, 89 ± 26 vs 69 ±13 (*P  = * 0.004), respectively. Levels of LDL and HDL cholesterol in plasma from patients with favorable outcome were significantly higher, 1.08 ± 0.43 vs 0.84 ± 0.36 (*P  = * 0.046) and 0.47 ± 0.15 vs 0.35 ± 0.09 (*P  = * 0.005), respectively. BNP levels at presentation were not significantly different between patients with favorable and adverse outcomes. APACHE II score was significantly higher in patients who died within 30 days. The mean length of stay in ICU was 11 ± 9 and 15 ± 8 days, before discharge and death, respectively.

**Table 1:** Characteristics of the patients with AHF.

	All patients	Favorable outcome	30-day mortality	
Characteristics	(n = 126)	(n = 98)	(n = 28)	*P*
Age (y)	69 ± 15	69 ± 15	69 ± 13	0.881
Sex, Female %, (n)	39 (49)	43 (42)	25 (7)	0.133
BMI	26 ± 5	27 ± 5	25 ± 7	0.396
Cardiovascular risk factors				
Hypertensive %, (n)	58 (73)	61 (60)	46 (13)	0.230
Diabete %, (n)	38 (48)	39 (39)	32 (9)	0.721
Dyslipidemia %, (n)	50 (63)	51 (49)	50 (14)	0.903
Obesity %, (n)	16 (20)	17 (16)	14 (4)	0.970
Heridity CD %, (n)	8 (10)	8 (8)	9 (2)	0.822
Previous cardiac disease				
Coronary artery disease %, (n)	52 (65)	52 (51)	50 (14)	0.978
Valvular heart disease %, (n)	30 (38)	29 (29)	32 (9)	0.943
Idiopatic dilated cardiomyopathy %, (n)	13 (16)	12 (12)	14 (4)	0.967
Admission diagnosis				
New onset of AHF %, (n)	41 (52)	47 (46)	22 (6)	0.031
Acute decompensation of CHF %, (n)	59 (74)	53 (52)	78 (22)	0.031
Acute coronary syndrome %, (n)	52 (65)	52 (51)	50 (14)	0.978
HF with reduced ejection fraction %, (n)	71 (89)	71 (69)	71 (20)	0.813
Clinical presentation				
Pulmonary oedema or Hypertensive AHF %, (n)	58 (73)	72 (71)	7 (2)	<0.001
Cardiogenic shock %, (n)	34 (43)	21 (21)	80 (22)	<0.001
Acute right-sided heart failure %, (n)	8 (10)	7 (7)	10 (3)	0.532
Admission medication				
ACE inhibitor %, (n)	32 (40)	36 (35)	18 (5)	0.112
Angiotensin receptor blocker %, (n)	2 (2)	2 (2)	0 (0)	0.911
Beta-blocker %, (n)	33 (41)	39 (38)	10 (3)	0.008
Diuretic %, (n)	91 (114)	93 (92)	80 (22)	0.090
Aldosterone antagonist %, (n)	25 (31)	24 (23)	30 (8)	0.615
Antiplatelet agent %, (n)	71 (89)	69 (67)	80 (22)	0.315
Vitamin K antagonist %, (n)	31 (38)	32 (31)	27 (7)	0.543
Statine	30 (37)	33 (32)	16 (5)	0.131
Early in-hospital management				
Inotropes administration				
Dobutamine %, (n)	22 (28)	14 (13)	53 (15)	<0.001
Norepinephrine %, (n)	19 (24)	9 (9)	53 (15)	<0.001
Epinephrine %, (n)	7 (9)	4 (4)	18 (5)	0.037
Ventilatory assistance %, (n)	27 (34)	21 (21)	46 (13)	0.017
Circulatory assistance %, (n)	8 (10)	6 (4)	14 (6)	<0.001
Laboratory data				
BNP (pg/ml)	1172 ± 1131	1154 ± 1090	1237 ± 1281	0.738
Na^+^ (mM)	136 ± 4	136 ± 4	136 ± 6	0.412
Creatinine (µM)	140 ± 79	145 ± 81	162 ± 89	0.229
C reactive protein (mg/l)	53 ± 69	50 ± 69	64 ± 72	0.340
Hb (g/dl)	13 ± 2	13 ± 2	13 ± 2	0.634
Glucose (mM)	7 ± 2	7 ± 2	7 ± 3	0.408
Bilirubin ( µM)	20 ± 15	18 ± 14	23 ± 16	0.121
Prothrombin ratio %, (n)	66 ± 26	67 ± 25	59 ± 25	0.119
LDL (g/l)	1.04 ± 0.43	1.08 ± 0.43	0.84 ± 0.36	0.046
HDL (g/l)	0.45 ± 0.15	0.47± 0.15	0.35 ± 0.09	0.005
TG (g/l)	1.05 ± 0.43	1.05 ± 0.43	1.05 ± 0.37	0.963
Admission vitals				
Mean blood pressure (mmHg)	84 ± 25	89 ± 26	69 ± 13	0.004
Heart rate (Bpm)	91 ± 28	89 ± 31	97 ± 16	0.343
Echocardiography				
LVEF %, (n)	35 ± 16	35 ± 14	35 ± 21	0.831
LVEF > 45% %, (n)	25 (31)	25 (24)	26 (7)	0.889
LV telediastolic diameter (mm)	61 ± 11	61 ± 11	62 ± 12	0.930
Pulmonary artery pressure (mmHg)	51 ± 16	52 ± 24	47 ± 21	0.453
Electrocardiography				
Sinus rhythm %, (n)	72 (90)	73 (72)	64 (18)	0.495
Radiography				
Cardiothoracic index (CI > 0.5)%	58 ± 6	58 ± 6	58 ± 6	0.584
APACHE II score, (IQR)	14 (13−16)	14 (12−15)	17 (13−20)	<0.001

*Heredity CD* occurrence of cardiovascular disease in first degree relatives, *AHF* acute cardiac failure, *CHF* cardiac heart failure, *HF* heart failure, *ACE* angiotensin-converting enzyme, *LDL* low density lipoproteins, *HDL* high density lipoproteins, *TG* triglycerides, *BMI* body mass index, *LVEF* left ventricular ejection fraction, *LV* left ventricular, *BNP* B-type Natriuretic Peptide concentration, *APACHE II* acute physiology and chronic health evaluation II scoring system, *IQR* interquartile range.

### NMR metabonomic profiling of patients’ plasmas

**Figure 2:**
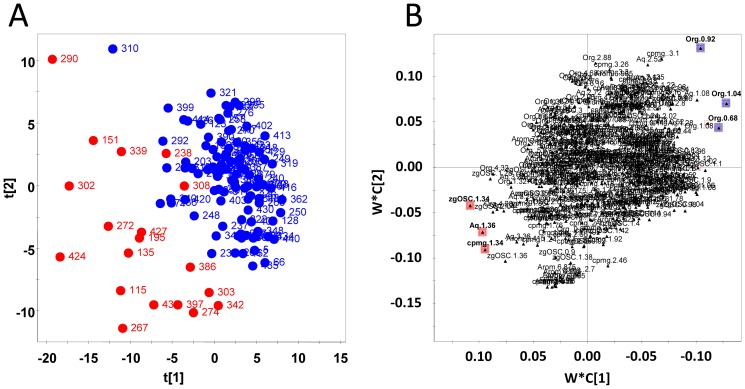
PLS-DA analysis of ^1^H NMR spectral data. **(A)** PLS-DA t1/t2 score plot derived from ^1^H NMR spectral data correlating to 86% of the Y-variance. The plot shows how well individuals separate whether it is a survivor (blue spot) or a deceased (red spot) individual (number is for the identification of individual). **(B)** PLS-DA weight plot (w*c_1_/w*c_2_). This plot of the X- and Y-weights (w* and c) shows how the ^1^H NMR spectral data correlate with class belonging i.e. the survival or 30-day mortality classes. Variables corresponding to the 6 first higher variable influence parameters have a colored box; red when there are positively correlated with 30-day mortality class and blue when there are positively correlated with survival class. The triangles were labeled by spectral data identification. List of these discriminating variables is in Table 2.

**Figure 3:**
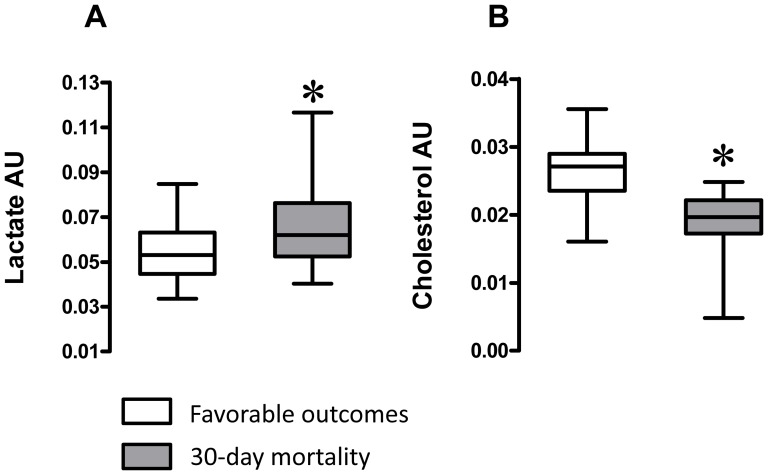
^1^H NMR signals intensities of lactate and cholesterol in plasmas. **(A)** Contribution of a ^1^H NMR characteristic signal of lactate at 1.36 ppm to the spectrum of aqueous fraction of the plasma extract (Aq spectrum). **(B)** Contribution of a ^1^H NMR characteristic signal of cholesterol at 0.68 ppm to the spectrum of lipid fraction of the plasma extract (Org spectrum). Values are expressed as arbitrary unit (AU). Lines at the middle for medians, boxes extend from 25^th^ to 75^th^ percentile, error bars for max and min values. Boxes: white, plasma from patients with favorable outcome; grey plasma from patients with 30-day mortality outcome. * is for Student’s *t* test p <0.05.

Data extracted from the ^1^H NMR spectra of the patient plasma were analyzed using partial least-square discriminant analysis. The score map (Figure 2A) shows separation of plasma samples based upon outcomes. Discriminant variables from each group are the ones with most extreme values on the loading plot (color spots; Figure 2B). Variables generating the highest influence on projection parameters with their corresponding metabolite are listed in Table 2. Increased signals intensities from cholesterol correlated positively with survival, whereas increased signals intensities from lactate correlated positively with 30-day mortality. Indeed, a significantly higher intensity of a characteristic signal of lactate (Figure 3A) relative to the total spectral intensity was observed in the aqueous fraction of plasma from the 30-day mortality group. Conversely, a significantly lower intensity of a characteristic signal of cholesterol (Figure 3B) relative to the total spectral intensity was measured in the lipid fraction of plasma from the 30-day mortality group.

**Table 2:** Metabolites contributing to NMR spectral variables with higher influence on projection parameters values.

Discriminating variables	Influence parameter	Contributing metabolites	Correlation Sign	
			30-Day mortality	Survival
Org_1.04 ppm	2.36	cholesterol	negative	positive
Org_0.68 ppm	2.22	cholesterol	negative	positive
Org_0.92 ppm	2.10	cholesterol	negative	positive
zg_1.34 ppm	1.98	lactate	positive	negative
cpmg_1.34 ppm	1.81	lactate	positive	negative
Aq_1.36 ppm	1.80	lactate	positive	negative

*Zg* indicates that the variable was extracted from Zg-spectra, *cpmg* indicates that the variable was extracted from cpmg-spectra, *Org* indicates that the variable was extracted from Org-spectra obtained on lipophylic fractions of plasma extracts, *Aq* indicates that the variable was extracted from Aq-spectra obtained on hydrophilic fractions of plasma extracts, *negative* correlation sign corresponds to lower signal intensity of the variable in the group, *positive* correlation sign corresponds to higher signal intensity of the variable in the group.

### Automated monitoring of venous plasma lactate and cholesterol

Quantitative analysis of plasma concentrations of lactate and total cholesterol were further determined by an enzymatic method on an aliquot of plasma sample. Lactate concentration was significantly higher in patients dead at 30 days that in patients alive at 30 days with 1.78 ± 1.32 (n = 28) vs 1.14 ± 0.40 mM (n = 98), (p<0.001), respectively. Conversely, total cholesterol concentration was significantly lower in patients who were dead at 30 days than in patients alive at 30 days with 2.37 ± 0.67 (n = 28) vs 3.23 ± 1.01 mM (n = 98), (p<0.001), respectively.

### Thirty-day mortality prognostic values of lactate, cholesterol and lactate to cholesterol ratio

**Figure 4:**
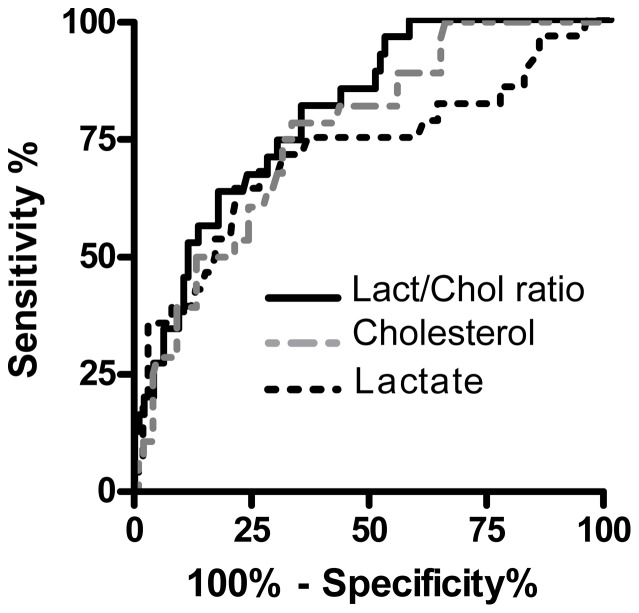
Receiver Operating Characteristic (ROC) curve analysis. ROC curves analysis of lactate, cholesterol and lactate to cholesterol ratio in relation with 30-day mortality in the training cohort.

Accuracies of lactate and total cholesterol concentrations to predict 30-day mortality were evaluated using ROC curve analysis (Figure 4 and Table 3). The accuracy was better for cholesterol (AUC: 0.76, *P* < 0.0001) than for lactate (AUC: 0.73, *P*  =  0.0003). Regarding the inverse relationship of lactate and cholesterol concentrations according to the outcome status of patients we investigated the strength of the lactate to cholesterol ratio (Lact/Chol) as prognostic index for 30-day mortality. The Lact/Chol ratio improved the accuracy, predicting 30-day mortality with AUC: 0.82 *(P* < 0.0001). The cutoff value corresponding to the maximum of the Youden index was at 0.4 with 82% sensitivity and 64% specificity.

**Table 3:** Receiver operating characteristic analysis results.

Variables	AUC	95% CI	*P*
Lactate	0.73	0.60−0.85	0.0003
Total Cholesterol	0.76	0.67−0.85	<0.0001
Lact/Chol ratio	0.82	0.73−0.89	<0.0001
APACHE II	0.75*	0.67−0.83	<0.0001

*AUC* area under ROC curve, *CI* confident interval, *APACHE II* acute physiology and chronic health evaluation II scoring system, *P* value tests the null hypothesis that the AUC really equals 0.5. * no significant difference between ROC curve of Apache II and Lact/Chol ratio, p = 0.389).

**Figure 5:**
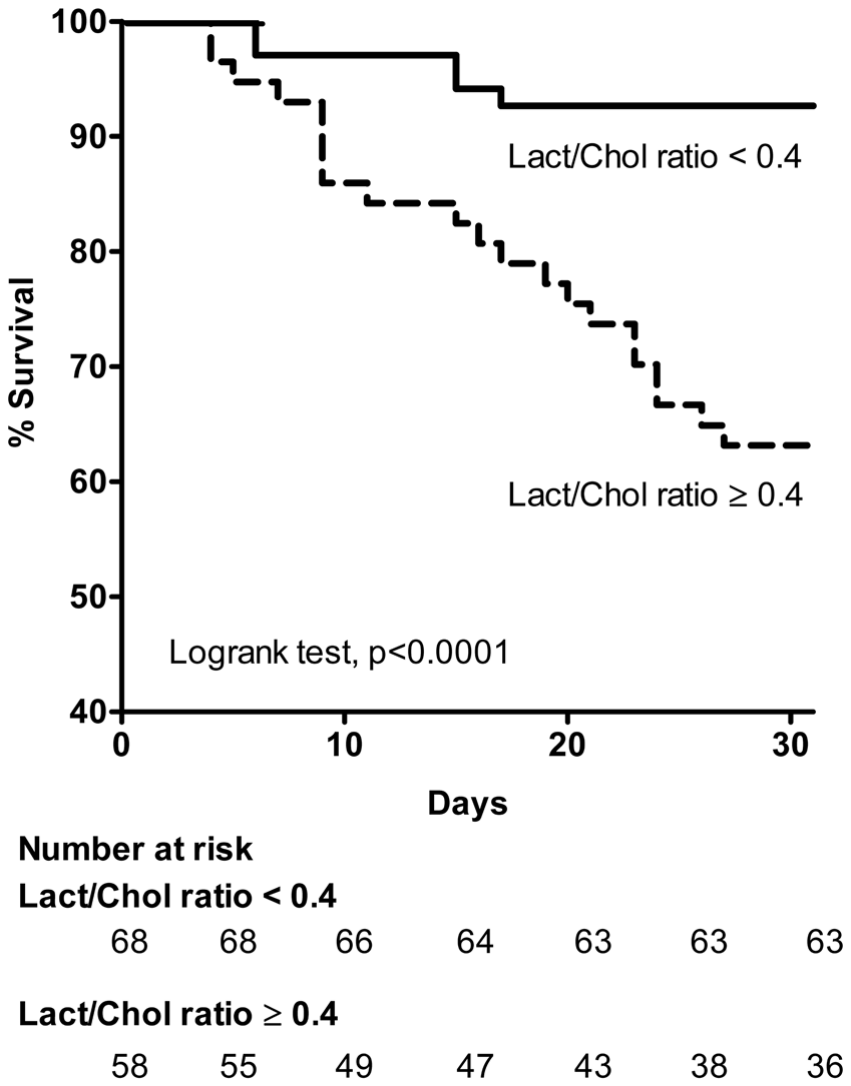
Survival analysis of the patients according to the cholesterol to lactate ratio. Kaplan−Meier curve estimation of the survival rate of the patients according to the cholesterol to lactate ratio at the cutoff value. A total of 126 AHF patients (training cohort) were included in this analysis based on **Cobas Mira+ automated enzymatic detection** of venous plasma lactate and cholesterol. Bold and dashed lines are for lactate/cholesterol ratio <0.4 and ≥ 0.4, respectively. Number at risk indicates over time the number of survival patients within each group based on lactate to cholesterol ratio (<0.4 and ≥ 0.4).

Survival analysis for the 2 subgroups generated with a Lact/Chol ratio cutoff value of 0.4 is presented under a Kaplan-Meier plot in Figure 5. The two survival curves differ significantly (p<0.0001) which confirmed the prognostic power for 30-day mortality of this index. The hazard ratio adjusted for age and sex of 30-day mortality in patients with Lact/Chol ratio ≥ 0.4 was 5.74 (95% CI, 2.13−15.48) compared with patients with Lact/Chol < 0.4 (Table 4).

**Table 4:** Univariate and multivariate Cox regression analysis of variables associated with 30-day mortality.

	Univariate Analysis*				Mutlivariate analysis =				
Variables	HR	95% CI		*P*	HR		95% CI		*P*
Age (y)	1.00	0.98−1.03		0.85					
Sexe, male = 1	1.99	0.84−4.74	0.12						
Clinical presentation, CS = 1	**8.84**	3.53−22.12	<0.001			**4.77**		2.79−17.50	0.001
MAP, < 74 mmHg = 1	2.47	0.85−7.14	0.098						
Admission diagnosis, ADCHF = 1	**2.54**	1.01−6.36	0.048						
LDL ,< 0.99 g/l = 1	3.81	0.97−8.30	0.052						
HDL, < 0.36 g/l = 1	**3.55**	1.26 −10.03	0.017						
APACHE II	**1.21**	1.12 −1.31	<0.001			**1.11**		1.04−1.20	0.006
Lact/Chol ratio, ≥ 0.40 = 1	**5.74**	2.13−15.48	<0.001			**3.59**		1.34−9.63	0.011
Lact/Chol ratio, 4^th^ quartile†	7.22	3.04−17.12	<0.001						

*CS* cardiogenic shock; *MAP* Mean blood pressure, *ADCHF* acute decompensation of CHF, *HR* hazard ratio, *CI* confident interval, *Lact/Chol* Lactate/cholesterol, *LDL* low density lipoproteins, *HDL* high density lipoproteins, *APACHE II* acute physiology and chronic health evaluation II scoring system.

* HRs of 30-day mortality outcomes adjusted for age and sex.

= stepwise regression, significance level of the model P < 0.0001.

† HR of patients in the highest quartile of Lact/Chol ratio (4^th^ quartile Lact/Chol  =  0.80 (95%CI, 0.66−1.90, n = 29); HR = 1, 1^st^ −3^rd^ quartile Lact/Chol  =  0.33 (95%CI, 0.29−0.36, n = 97)).

Statistically significant values are in bold.

### Univariate and multivariate predictors of 30-day mortality

Patients with AHF were divided into 2 groups based on each variable associated with 30-day mortality. For continuous variables, the cutoff level was calculated by receiver operating characteristics analysis to detect 30-day mortality and 30-day mortality prediction was evaluated by Cox proportional hazards ratio analysis (Table 4). The cutoff level was determined as 74 mmHg for MAP, giving a sensitivity of 71.4% and a specificity of 71.1%; 0.99 g/l for LDL, giving a sensitivity of 80% and a specificity of 52.1; 0.36 g/l for HDL, giving a sensitivity of 60% and a specificity of 77.5. By univariate Cox proportional hazards regression analysis, clinical presentation with cardiogenic shock (CS), a high value of Lact/Chol ratio, low level of HDL, acute decompensation of CHF (ADCHF) and APACHE II were all significant predictors of 30-day mortality (Table 4). By stepwise multivariate Cox analysis only CS, APACHE II and Lact/Chol ratio of > 0.4 were significant independent predictors of 30-day mortality with hazards ratios of 4.77, 1.11 and 3.59, respectively. The c-statistic for APACHE II capability in predicting 30-day mortality was 0.75 which was lower but not significantly different of 0.82 measured for Lact/Chol ratio (Table 3).

### Mortality prediction stratified by the combination of cardiogenic shock and lactate to cholesterol ratio

**Figure 6:**
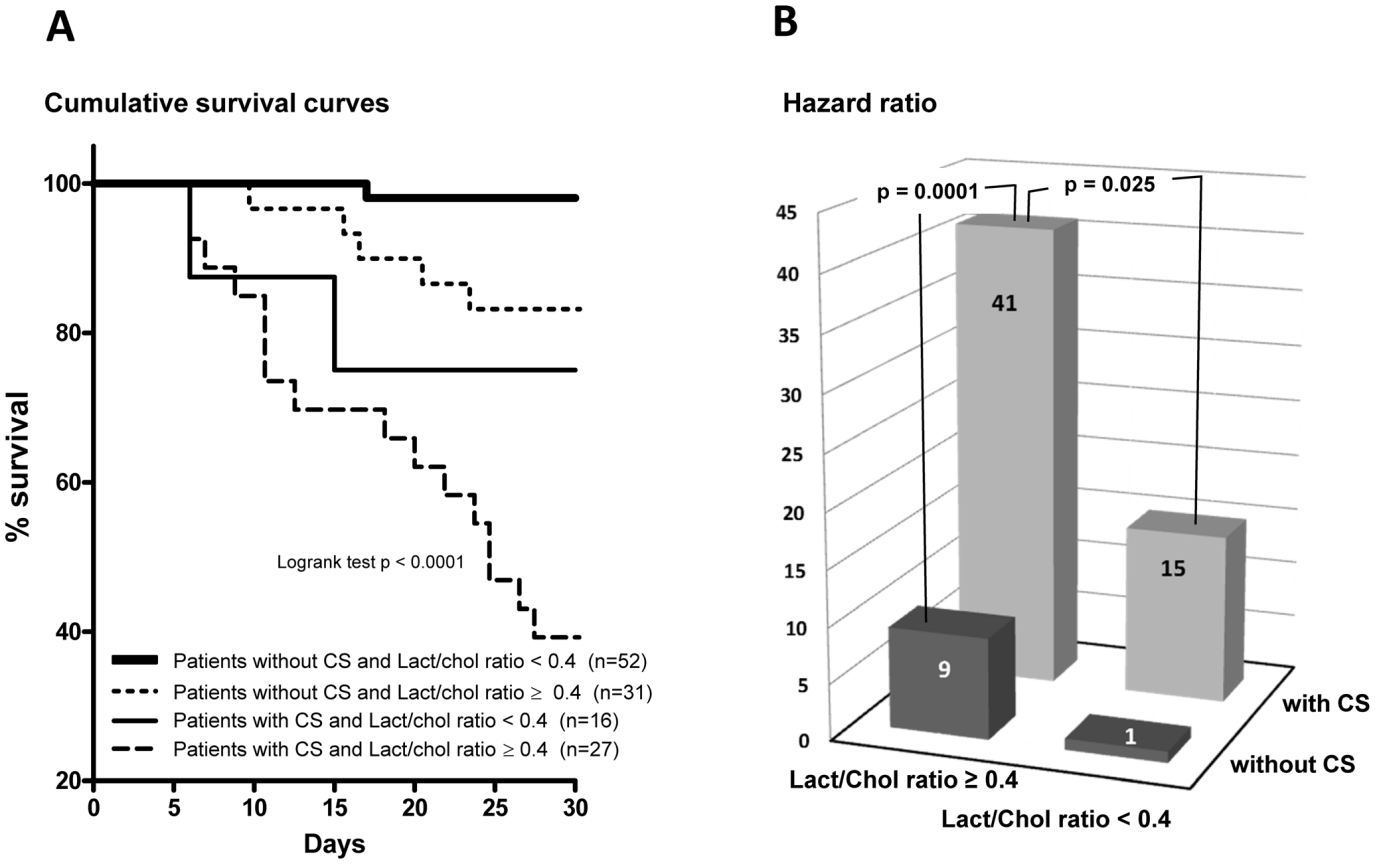
Survival analysis of the training cohort by the combination of clinical presentation and lactate to cholesterol ratio. (A) Kaplan Meier survival curves for patients with AHF of the training cohort stratified into 4 groups based on the combination of clinical presentation (with or without cardiogenic shock (CS)) and the cutoff level of lactate to cholesterol ratio (Lact/Chol ratio). (B) Usefulness of 30-day mortality prediction using Lact/Chol ratio and clinical presentation. Hazard ratio with referent group (i.e. without CS and a Lact/Chol ratio < 0.4) for «Lact/Chol ratio ≥ 0.4 — without CS», 9 (p = 0.015); for « Lact/Chol ratio < 0.4 — with CS», 15 (p = 0.0014) and for « Lact/Chol ratio ≥ 0.4 — with CS», 41 (p<0.0001).

Patients were stratified into 4 groups based on CS status and cutoff level of Lact/Chol ratio (Figure 6). Compared with patients who had not presented a CS and a plasma Lact/Chol ratio < 0.4, patients who had presented a CS and a plasma Lact/Chol ratio ≥ 0.4 had a 41 fold higher 30-day mortality risk (p < 0.0001) with a median survival of 24 days. Furthermore, in patients who had presented a CS, the HR of 30-days mortality risk in patients with plasma Lact/Chol ratio ≥ 0.4 was 3.26 compared with that in patients with plasma Lact/Chol ratio < 0.4 (P  =  0.018).

### Validation cohort

**Figure 7:**
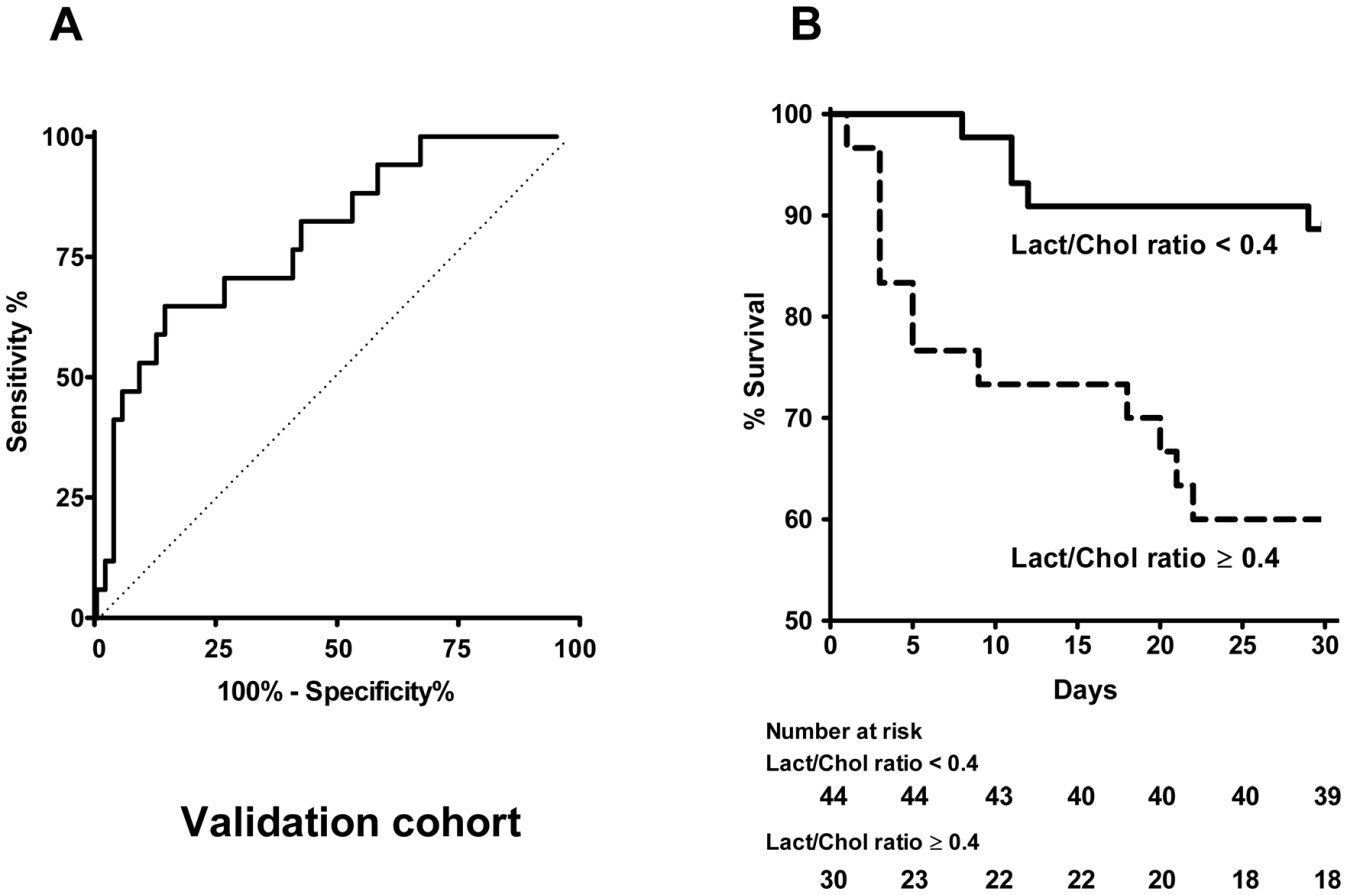
Predictive power of the Lact/Chol ratio in the validation cohort. Patients of the validation cohort were included (n = 74) in this analysis: with favorable outcome (n = 57) and with 30-day mortality outcome (n = 17). (A) Receiver Operating Characteristic (ROC) curve analysis of lactate, cholesterol and lactate to cholesterol ratio in relation with 30-day mortality), AUC  =  0.76 (95% CI, 0.65−0.85, p<0.0001). (B) Kaplan–Meier curve estimation of the survival rate of the patients according to the cholesterol to lactate ratio at the cutoff value. Bold and dashed lines are for lactate/cholesterol ratio <0.4 and ≥ 0.4, respectively. Number at risk indicates over time the number of survival patients within each group based on lactate to cholesterol ratio (<0.4 and ≥ 0.4).

An independent cohort of 74 consecutive patients admitted for AHF (Table 5) was enrolled to validate capability of Lact/Chol ratio for predicting 30-day mortality. The ROC analysis determined an AUC of 0.76 (95% CI, 0.65−0.85; p<0.0001) (Figure 7A). When applying the predefined cut-off value of 0.4 as criterion value, coordinates of the ROC curve were 82% for sensitivity and 54% for specificity which are similar to the characteristics obtained with the training cohort. The survival curves of subgroups of patients generated with a Lact/Chol ratio cutoff value of 0.4 differed significantly (p<0.005) (Figure 7B). In the validation cohort Lact/Chol ratio, CS status, MAP and sodium level was associated with 30-day mortality. The cutoff level of sodium was determined as 126 mM giving a sensitivity of 76% and a specificity of 58%. The hazard ratio of 30-day mortality in the subgroup of patients with higher Lact/Chol ratio was 5.50 and 5.33 when adjusted for age, sex and CS (Table 6). These findings are in good accordance with data reported for the training cohort.

**Table 5:** Characteristics of the patients of the validation cohort.

			All patients			Favorable outcome		30-day mortality	
Characteristics			(74)			(57)		(17)	*P*
Age (y)			72 ± 15			71 ± 16		73 ± 16	0.648
Sex, Female %, (n)			30 (22)			32 (18)		24 (4)	0.763
BMI			26 ± 5			26 ± 5		27 ± 7	0.749
Cardiovascular risk factors									
Hypertensive %, (n)			61 (45)			61(35)		59 (10)	1.000
Diabete (%)			34 (25)			33 (19)		35 (6)	1.000
Dyslipidemia %, (n)			38 (28)			35 (20)		47 (8)	0.404
Obesity %, (n)			21 (16)			23 (13)		18 (3)	0.749
Clinical presentation									
Congestive heart failure %, (n)			44 (33)			44 (25)		47 (8)	1.000
Cardiogenic shock %, (n)			15 (11)			9 (5)		35 (6)	0.014
Pulmonary oedema or Hypertensive AHF%,(n)			27 (20)			32 (18)		12 (2)	0.130
Admission medication									
ACE inhibitor %, (n)			28 (21)			30 (17)		24 (4)	0.763
Angiotensin receptor blocker %, (n)			4 (3)			5 (3)		0 (0)	1.000
Beta-blocker %, (n)			30 (22)			32 (18)		24 (4)	0.763
Diuretic %, (n)			89 (66)			91 (52)		82 (14)	0.090
Aldosterone antagonist %, (n)			10 (7)			5 (3)		24 (4)	0.044
Antiplatelet agent %, (n)			63 (47)			63 (36)		65 (11)	1.000
Early in-hospital management									
Inotropes administration									
Dobutamine%, (n)			16 (12)			12 (7)		30 (5)	<0.131
Norepinephrine %, (n)			18 (13)			10 (6)		42 (7)	<0.008
Epinephrine %, (n)			12 (9)			7 (4)		30 (5)	0.037
Ventilatory assistance %, (n)			46 (34)			37 (21)		78 (13)	0.017
Circulatory assistance %, (n)			13 (10)			7 (4)		36 (6)	<0.001
Laboratory data									
BNP (pg/ml)			1378 ± 1268			1354 ± 1311		1455 ± 1147	0.301
Na^+^ (mM)			136 ± 5			137 ± 4		134 ± 7	0.049
Creatinine (µM)			151 ± 94			147 ± 94		167 ± 93	0.179
C reactive protein (mg/l)			76 ± 75			75 ± 73		75 ± 86	0.676
Glucose (mM)			8 ± 4			8 ± 3		11 ± 7	0.086
Bilirubin (µM)			20 ± 15			20 ± 16		18 ± 12	0.607
Prothrombin ratio (%)			67 ± 20			67 ± 19		64 ± 21	0.649
Admission vitals									
Mean blood pressure (mmHg)			80 ± 15			82 ± 14		73 ± 15	0.021
Heart rate (Bpm)			87 ± 28			89 ± 31		88 ± 31	0.852
Echocardiography									
LVEF (%)			36 ± 14			38 ± 15		31 ± 12	0.089
LVEF > 45%, (n)			27 (20)			30 (17)		18 (3)	0.372
Electrocardiography									
Sinus rhythm %, (n)			57 (42)			63 (36)		35 (6)	0.053
				

*ACE* angiotensin-converting enzyme, *LDL* low density lipoproteins, *HDL* high density lipoproteins, *TG* triglycerides, *BMI* body mass index, *LVEF* left ventricular ejection fraction, *LV* left ventricular, *BNP* B-type Natriuretic Peptide concentration. Numbers in parenthesis indicate number of patients.

**Table 6:** Cox regression analysis for 30-day mortality in the validation cohort.

	Univariate Analysis*			Mutlivariate analysis^ = ^		
Variables	HR	95% CI	*P*	HR	95% CI	*P*
Age (y)	1.00	0.97−1.04	0.636			
Sexe, male = 1	0.83	0.27−2.57	0.752			
Clinical presentation, CS = 1	**8.53**	2.45−29.66	<0.001	2.70	0.93−7.64	0.062
Lact/Chol ratio, ≥ 0.4 = 1	**5.50**	1.77−17.08	0.003	**5.33**	1.17−23.98	0.031
MAP, < 74 mmHg = 1	**3.15**	1.16−8.52	0.024			
Serum sodium, <136 mM	**3.04**	1.08−8.37	0.032			

* HRs of 30-day mortality outcomes adjusted for age and sex. ^ = ^ Cox proportional-hazards stepwise regression with clinical presentation CS, Lact/Chol ratio, MAP and serum sodium as predictor variables, significance level of the model P  =  0.002. *CS*, cardiogenic shock. *HR*, hazard ratio. *CI*, confident interval. Figures in bold are statistically significant.

## Discussion

We have shown that in patients with AHF, a high level of venous plasma lactate concentration, a low level of total cholesterol concentration and a high Lact/Chol ratio are predictors of 30-day mortality. In other words, the Lact/Chol ratio measured at the admission is an independent short-term prognostic 30-day indicator of mortality.

This study was first conducted without any *a priori* analysis to find potent correlations between ^1^H NMR spectra and 30-day outcomes. We chose to use ^1^H NMR spectroscopy based metabolic profiling because of its efficiency to indentify plasma metabolites as reliable biomarkers of miscellaneous pathologies [15], [18]. Statistical analysis of ^1^H NMR spectral data revealed that signals corresponding to venous lactate and total cholesterol were strongly associated with patients’ death within 30 days following admission. This was further confirmed by a second analysis of the plasma drawn using autoanalyzer techniques for quantification of lactate and cholesterol. The observed rise in venous blood lactate concentration in patients who died reflects inadequate organ perfusion of peripheral tissues leading to enhanced anaerobic glycolysis. It also assesses the severity of shock situations [20], [21]. In chronic heart failure, serum lactate was not predictive of the severity of heart failure in a population of cardiac transplant candidates [22].

Plasma cholesterol level is the result of a balance between absorption by the digestive system and hepatic synthesis. Lower total cholesterol levels have been associated with increased in-hospital mortality of patients with various diseases, including miscellaneous heart diseases, and was proposed as one of the first signs of forthcoming deterioration of a preexisting disease [23]. Indeed, hypocholesterolemia is a signal of disease state in a number of pathologies [24]. These include hepatic failure, hyperthyroidism, malnutrition, poor digestive absorption, inflammatory syndromes, trauma and infectious diseases [25]. Hypocholesterolemia is also associated with high perioperative mortality in patients supported by a left ventricular assistant system [26] and with increased mortality in advanced or acute heart failure patients [27]. In agreement with this, total cholesterol, HDL and LDL cholesterol concentrations were significantly lower in patients who died.

We aimed to find an index that would be easy to determine based on the venous cholesterol and lactate concentration. Analysis of receiver operating curves demonstrates that Lact/Chol ratio with an AUC of 0.82 has a higher discriminatory power for 30-day mortality than lactate or cholesterol concentrations alone. In this study, patients with a Lact/Chol ratio superior to the best cutoff value (0.4) had a 6-fold higher risk for 30-day mortality. Similar results were obtained with the independent validation cohort *i.e*. AUC  =  0.76 and HR  =  5.5. We compared the discrimination power of the Lact/Chol ratio with the APACHE II scoring system, the world's most widely used outcome scoring systems [28]. Determination of APACHE II score required several patient characteristics including comorbid conditions and 12 physiological and laboratory parameters obtained during the 24 first hours following ICU admission. In this study we observed similar discriminatory power for APACHE II score and Lact/Chol ratio. Indeed, APACHE II scoring system has been developed as a tool for comparing the outcomes of acute diseases in critically ill patients across multiple intensive care units [29]. It is recognized that APACHE II scoring varies considerably with the underlying diagnosis from country to country and has a high inter-observer variation. Thus, because of our focused screen for AHF prognosis biomarkers, it was expected to identify an objective parameter which performs well as predictor of 30-day mortality. The Lact/Chol ratio predicting value was similar to the one gained from the complex APACHE II score calculation which includes subjective parameters [28]. The majority of patients with a 30-day mortality outcome had a CS at admission and had the higher risk of death. High Lact/Chol ratio in these patients was associated with a large increase of the 30-day mortality risk.

Although it has been well established that BNP measurements at admission are biomarkers for heart failure and the long term prognosis of patients, its short term prognostic value is less accurate in the context of sepsis [30] or chronic kidney disease [31]. Several reports have focused on the prognostic role of serial measurements of BNP serum levels or percentage of BNP reduction to improve the short- or long-term prognostic value of BNP [32], [33], [34]. AUC of ROC curve for BNP level at admission plotted to test for short-term prognosis gives a poor predictive value of 0.64 compared to NT-proBNP (0.78) for 90-day mortality [5]. In agreement with these reports, BNP plasma levels measured at admission were not a predictor for 30-day mortality. Clinical presentation with CS, diagnosis of acute decompensation of CHF and Lact/Chol ratio remain variables significantly associated to 30-day mortality in the multivariate Cox regression analysis.

The prognostic utility of other blood biomarkers such as copeptin, MR-proADM and troponin has been evaluated in patients with acute heart failure. Thus, based on the analysis of the large multicenter database of patients hospitalized for heart failure in the United States (ADHERE registry) [35], high troponin (cTnI and cTnT) levels were associated with increased in-hospital mortality [8]. The adjusted odds ratio for in-hospital death among patients with positive troponin test and serum creatinine level of less than 2.0 mg per deciliter was 2.55. By using the same database and CART method, blood urea nitrogen was found as the most significant predictor of in-hospital mortality [36]. More recently, the prognosis utility of copeptin, the C-terminal segment of preprovasopressin, and midregion proadrenomedullin (MR-proADM) were evaluated in an acute heart failure (BACH) study [37]. In a multivariate model including the BNP, NT-proBNP, copeptin and MR-proADM as independent prognostic factor, the MR-proADM and copeptin were the strongest for predicting 90-day mortality in patients presenting with acute dyspnea. The AUCs for 30-day mortality outcome for BNP, MR-proBNP, MR-proADM and copeptin was 0.55, 0.64, 0.74 and 0.73 in patient with AHF from BACH study [38], [39]. Patients in the highest quartile of copeptin had increased 90-day mortality with a HR of 3.8 [40]. In this study, we did not evaluate these new prognostic makers; however, as a matter of comparison, the AUC for 30-day mortality for Lact/Chol ratio was 0.82 and patients in the highest quartile of Lact/Chol had a HR of 7 for 30-day mortality. This suggests that Lact/Chol ratio has a better or similar predictive performance for 30-day mortality to copeptin and MR-proADM.

Generally, physicians substantially over estimate risk, resulting in over-utilization of critical care resources [41]. Thus, many studies have been recently conducted to improve or develop predictive models for in-hospital mortality [39], [42]. These models use commonly available clinical variables to calculate a mortality risk score. Candidate predictor variables are *a priori* selected based on literature, clinical relevance and general availability at time of presentation. In the present study, using a without a *priori* metabolomics analysis of plasma we identified Lact/Chol as an independent predictor variable. Because lactate and cholesterol assays are routine laboratory tests it would be valuable to evaluate the Lact/Chol ratio as a candidate variable in future predictive models of in-hospital mortality risk determination. Furthermore, determining whether prospective application of the risk prediction score will adequately affect patient care and clinical outcomes should be the topic of future study.

Serial measurements of lactate and cholesterol plasma levels during hospitalization were not obtained in this study but our data suggested that they could be also used as an efficiency index for the medical survey. However, this severity index needs further testing in a large scale, multi-center study to confirm an optimum threshold value. Clinical use of such an index would be of major interest at hospital admission of acute heart failure patients.

In conclusion, the present study underlines the clinical utility of the Lact/Chol ratio in patients hospitalized with congestive heart failure. A large multicentric study is warranted to assess the leverage of plasma Lact/Chol ratio on 30-day mortality risk models which remain useful quantitative guidance for decision making in heart failure care.

### Limitations of this study

This is a monocentric study carried out with patients hospitalized in French hospital where all patients with acute heart failure are addressed to a specialized cardiac intensive care unit (USIC, Unité de Soins Intensifs Cardiologiques). Thus, patients with the most severe cardiogenic shock are included in the cohort whereas in other countries medical care management directs that these patients are usually maintained in an emergency department. This explains the mortality rate measured in this study which is similar to 28% in-hospital mortality rate observed in EFICA (French study of acute heart failure) but higher than the 4% mortality rate measured in the ADHERE study [36]. Patients with chronic liver failure or who have had an acute heart failure episode in the 6 months before admission were not included in the study. Unfortunately, the size of our cohort did not allow for a deeper categorization of patients, in particular for acute decompensated CHF patients.
